# Designing translational workforce development programs using the research knowledge and transfer utilization framework

**DOI:** 10.1017/cts.2026.10789

**Published:** 2026-07-13

**Authors:** Alexandria A. Carey, Elizabeth C. Dunn, April N. Braxton, Jennifer Moses, Ramzi G. Salloum

**Affiliations:** 1 Clinical and Translational Science Institute, University of Florida, Gainesville, FL, USA; 2 Health Outcomes and Biomedical Informatics, University of Florida, Gainesville, FL, USA

**Keywords:** translational workforce development, clinical research professionals, knowledge transfer and utilization, program administrators, multimodal role-aligned training model

## Abstract

Translational Workforce Development (TWD) is critical for Clinical and Translational Science Award (CTSA) hubs preparing Clinical Research Professionals (CRPs) to conduct high-quality research. Yet programs struggle to reflect the practical demands of CRP roles, and generalized training often leads to disengagement and limited transfer of learning into practice. Using dissemination science principles, we applied findings from a single-site CRP needs assessment and the Research Knowledge Transfer Utilization (RKTU) framework to inform a redesigned approach to TWD. Survey data (*n* = 65) were analyzed to identify training needs, contextual factors, preferred formats, motivators, and knowledge broker and champion roles in supporting engagement and sustained knowledge use. CRPs described inconsistent onboarding, limited structured support, and challenges aligning existing training with real-world workflows. Respondents expressed interest in practice-based, role-aligned learning delivered through blended formats such as on-demand modules, hybrid workshops, and hands-on simulation. Motivators included career pathways, community connection, and protected time, while workload and system complexity were key barriers. Despite these challenges, CRPs demonstrated readiness to learn when supported by clear expectations and accessible resources. The resulting model provides administrators with a scalable, context-informed approach for co-creating TWD programs embedded within organizational systems and aligned with the realities of clinical research practice.

## Introduction

Translational Workforce Development (TWD) is built around a simple idea: accelerating research discoveries into health solutions [1]. It does this by preparing clinical research professionals (CRPs) to protect participants, ensure data integrity, and support high-quality research [1]. Effective training must align with the realities of daily CRP work, yet many TWD programs rely on generalized, one-time modules that may be perceived to be disconnected from actual study activities [2–6]. When training lacks context or practical relevance, CRPs often complete it without building meaningful competency, which limits program impact and leaves both learners and administrators dissatisfied [2,4].

Evidence shows that CRPs benefit most from role-specific, applied practice-based learning (APBL) that connects theory to the operational demands of clinical research [6,7]. Approaches built solely on one-way knowledge transfer (KT) often break down because they overlook local workflow barriers, organizational climate, and the need for accountability structures [2–4]. Dissemination and Implementation (D&I) Science offers useful guidance for designing context-informed training that promotes adoption and sustained use [8]. In this regard, the *Conceptual Framework for Research Knowledge and Transfer Utilization* (RKTU) is particularly helpful because it emphasizes knowledge utilization (KU), relational factors such as trust, and participatory design through knowledge transfer and exchange (KTE) [2–4]. Within the framework, knowledge brokers (KBs) and champions play key roles in supporting CRPs as they interpret new information, navigate local systems, and adopt evidence-based practice (EBP) [2–4].

This Special Communication applies RKTU principles to inform TWD strategies using findings from a single-site CRP needs assessment. By translating these findings into a practical model, our goal is to provide Clinical and Translational Science Award (CTSA) hubs and academic health centers (AHCs) with an evidence-informed guide for creating TWD programs that CRPs are more likely to use, strengthening workforce capacity and supporting national translational science priorities [1,4,8].

## Methods

### Conceptual framework

**Figure 1.**
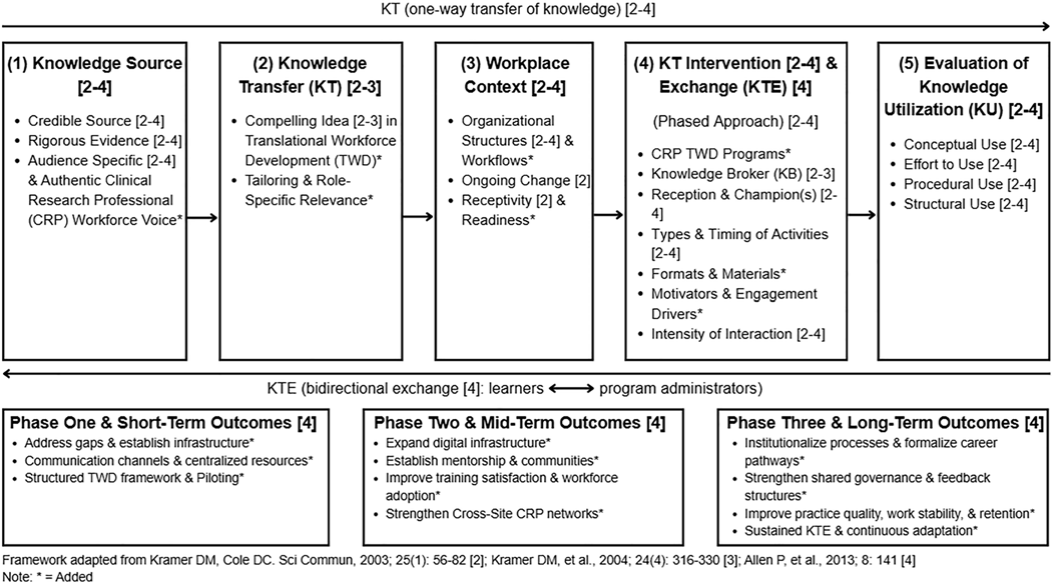
Figure 1 long description. Research knowledge and transfer utilization framework.

An adapted version of the RKTU framework guided the design, analysis, and interpretation of this needs assessment. Core components (e.g., knowledge sources, workplace context, KT/KTE, and KU evaluation) were retained and operationalized to reflect CTSA hub structures and TWD priorities. Adaptations emphasized source creditability, local workflows and systems, organizational conditions, and learner-informed KT/KTE approaches to align with clinical research environments. These components informed survey item development, guided framework-based analytic categorization, and supported interpretation of training needs, barriers, and implementation strategies across domains (Figure 1) [2–4]. The adapted framework provided a structured approach for linking individual learner perspectives with organizational context to inform TWD program design.

### Survey design

This needs assessment was conducted by a CTSA hub in the United States southeastern region, a National Center for Advancing Translational Sciences (NCATS) mission-aligned entity responsible for advancing CRP training and research quality [1]. Investigators developed the survey using relevant literature, existing CRP curricula, and the strategic priorities of a single AHC CTSA hub’s TWD program (see Supplementary Material) [9–11]. The survey, delivered via Qualtrics, aimed to capture CRP training preferences, motivations, and contextual factors influencing engagement and received institutional review board (IRB) exemption [9–12].

The final survey included optional fixed-choice and open-ended questions across six domains: (1) workplace characteristics; (2) training and education; (3) role-and organization-level challenges; (4) preferred training formats and motivators; (5) interest in CRP community activities; and (6) recommendations for improving training [2–4].

### Survey distribution

The survey was distributed in Spring 2024 through the CTSA hub ListServ [13] and direct email to CRPs across two member AHCs, affiliated clinical sites, and participating academic units (approximately 750 individuals). To promote candid responses, the survey was administered anonymously and reviewed for potential duplication. Two reminder emails were sent to encourage participation, and consistent with the IRB exemption, no remuneration was provided for completing the survey.

### Analysis approach

Analyses were conducted by members of the research team, who are part of the CTSA hub leadership with expertise in clinical research workforce development. Quantitative items were summarized using descriptive statistics to characterize onboarding experiences, preferred training formats, motivators, and funding patterns. Multi-response selections were tallied to identify the most frequently endorsed needs. Open-ended responses were analyzed using a framework-informed thematic approach aligned with key RKTU domains. Coding focused on descriptions of misalignment between training and role expectations, system-level challenges, workload constraints, and relational factors that shaped how CRPs engage with TWD opportunities.

#### Dissemination mapping

Survey findings were mapped to key dissemination activities, including stage-based planning, tailored messaging, and organizational embedding strategies [2–4]. This process clarified which messages resonated with CRPs, where multiple KT/KTE approaches were needed, and how insights could be integrated into existing structures such as onboarding, competency frameworks, and professional development (PD) programming [14].

## Results

### Component 1: Knowledge source [2,3]

#### Credible source

Conducting the needs assessment through a CTSA hub strengthens confidence in the credibility and relevance of the TWD findings [1–3,13].

#### Workforce characteristics and voice

Sixty-five CRPs completed the survey, including coordinators (*n* = 38; 58%), administrators (*n* = 16; 25%), assistants (*n* = 3; 5%), a regulatory analyst (*n* = 1; 2%), a data specialist (*n* = 1; 2%), and investigators (*n* = 6; 9%). Half (*n* = 31; 48%) reported more than ten years of research experience, indicating a seasoned sample well positioned to comment on training relevance and gaps. Narrative responses described the real pressures of daily research work (e.g., *“taking on the workload of three research coordinators”)* and provided an authentic account of how training is experienced in practice (e.g., “*jumped into my role without guidance”).* These examples provide essential context for interpreting gaps through the RKTU framework [2,3].

#### Rigorous evidence

The breadth of roles, responsibilities, and experience levels represented in the sample provided a solid foundation for identifying shared and role-specific needs. Respondents offered detailed examples of onboarding quality (e.g., described as *“mostly useless”* or involving being *“given an outdated paper handbook”*), workflow challenges (e.g., *“so many layers of regulatory [process] makes it so challenging to meet deadlines”* and *“takes ages to complete tasks”*), and areas where enhanced training could better support daily responsibilities (e.g., *“it would be beneficial to the research enterprise to have sustainable training and professional development opportunities to aid in hiring and retention”)* [1–3].

### Component 2: Knowledge transfer [2,3]

#### Workforce development compelling idea

Survey findings indicated that 71% of CRP respondents benefit most from training that clearly connects to the responsibilities they conduct each day (practice-based approaches). Respondents emphasized the need for task-level workflow training, streamlining, and role-specific scenarios, such as step-by-step walkthroughs for collaborating with regulatory authorities, standard operating procedure (SOP) coordination, data capture and management, research navigation, and stakeholder communication. Additionally, 71% of respondents expressed a desire for training that helps them understand how individual workflows fit within the study lifecycle, identify opportunities to improve processes, and recognize their impact on the broader clinical research ecosystem, thereby strengthening awareness of role importance, enhancing accountability, and boosting confidence in performing core responsibilities [2–4].

#### Audience-specific tailoring

CRPs described training needs that varied across roles. Coordinators, administrators, investigators, and technical staff each identified distinct combinations of skill areas that would strengthen their workflow rigor. Participants noted that role-specific examples and content tailored to their functional responsibilities and experience levels would help them apply training more effectively [1,4].

#### Role-specific relevance

CRPs identified several priority areas for onboarding (*n* = 44; 68%), including (1) simulation and role-playing (*n* = 31; 70%); (2) a standardized, comprehensive approach with role-specific training on responsibilities (*n* = 30; 68%); (3) institutional resources and requirements (*n* = 28; 64%); (4) precepting and shadowing (*n* = 24; 55%); (5) mentoring and coaching (*n* = 22; 50%); and (6) resource navigation (*n* = 19; 43%).

Respondents also reported interest in ongoing competency-based education (CBE) that aligns with expertise-leveled clinical research [15] (*n* = 56; 86%), in addition to their usual informed consent and Good Clinical Practice (GCP) required trainings, with comments such as we need *“more required trainings based on where we need it.”* CBE in order of priority include: (1) study and site management (*n* = 39; 70%); (2) data management and informatics (*n* = 37; 66%); (3) leadership and professionalism (*n* = 37; 66%); (4) communication and teamwork (*n* = 35; 63%); (5) ethical and participant safety considerations (*n* = 28; 50%); (6) clinical study operations (*n* = 27; 48%); (7) investigational product development and regulation (*n* = 15; 27%); and (8) scientific concepts and research design (*n* = 10; 18%) [15] (see Table 1). These needs evolved with experience level; newer CRPs prioritizing foundational guidance and more experienced CRPs expressing interest in advanced or system-level competencies [4] (see Supplementary Material).

**Table 1.** Priority training needs matrix (per role) Table 1 long description.

Priority training needs	Coordinator (*n* = 38)	Administrator (*n* = 16)	Investigator (*n* = 6)	Other (*n* = 5)
**Onboarding**				
Institution policies, SOPs, & guides	●	●●	●	●●
Institution resources/requirements	●●●	●●	●●●	●●
Local/regional resource navigation	●●	●●	●	●●
Mentoring/coaching	●●	●●	●	●●
Precepting/shadowing	●●	●●●	●	●●●
Simulation/role-playing/demonstration	●●●	●●●	●●●	●●●
Standardized role-specific CBE framework	●●●	●●	●	●●●
**Ongoing professional development**				
Scientific concepts & research design	●	●	●●	●●
Fundamental	●	●●●	●●	●
Skilled	●	●	●	●●
Advanced	●	●	●	●●●
Ethical & participant safety considerations	●●	●●	●●	●●
Fundamental	●●	●	●●●	●
Skilled	●●	●●●	●	●●●
Advanced	●●	●●	●	●●●
Investigational products development & regulation	●	●	●●	●
Fundamental	●●	●	●●●	●
Skilled	●	●	●	●
Advanced	●	●●	●	●
Clinical study operations (GCP)	●●	●●	●●	●●
Fundamental	●●	●	●●●	●
Skilled	●●	●●●	●	●●●
Advanced	●●	●●	●●●	●●●
Study & site management	●●●	●●	●●●	●●●
Fundamental	●●●	●	●●●	●●●
Skilled	●●●	●●●	●●●	●●●
Advanced	●●●	●●	●●●	●●●
Data management & informatics	●●●	●●	●●	●●●
Fundamental	●●●	●	●●●	●●●
Skilled	●●●	●●●	●	●●●
Advanced	●●	●●	●●●	●●●
Leadership & professionalism	●●●	●●	●●	●●●
Fundamental	●●	●	●●●	●●●
Skilled	●●●	●●●	●●●	●
Advanced	●●	●●●	●	●●●
Communication & teamwork	●●	●●	●●●	●●
Fundamental	●●	●	●●●	●
Skilled	●●●	●●●	●●●	●●●
Advanced	●●●	●●	●●●	●●●

Top Need = ●●● (67 – 100%); Moderate Need = ●● (34 – 66%); Lowest Need = ● (0 – 33%).

Sp. = Specialist; CBE = Competency-Based Education; EHR = Electronic Health Record; CTMS = Clinical Trial Management System; SOP = Standard Operating Procedures; GCP = Good Clinical Practice.

*Note:* The “Other” column reflections from Data Specialist, Regulatory Analyst, and Research Assistants. Dot priorities represent aggregate onboarding and professional development needs, with role-level adjustments where coded responses show stronger emphasis. Because roles have small Ns, differences should be interpreted cautiously-as guidance for program design rather than statistical inferences. The ongoing professional development domains are structured to current national clinical trial competencies (Joint Task Force for Clinical Trial Competencies) (Multi-Regional Clinical Trials, 2026 [15]).

### Component 3: Workplace context [2,3]

#### Organizational structures and workflows

Respondents described working within a large, multifaceted academic health environment where workflows and processes vary across departments [13]. This variability often created uncertainty about procedures and current expectations and contributed to inefficiencies in day-to-day research operations. CRPs reported that navigating inconsistent departmental processes was time-consuming and frequently required informal workarounds, limiting their ability to consistently apply training in practice. Clearer guidance and aligned workflows were viewed as essential for supporting effective training transfer and increased confidence in performing core research responsibilities [4].

#### Ongoing change

CRPs reported frequent changes to systems and policies that required ongoing adjustments in their workflow. Because information is often distributed across multiple sources, staying current was described as challenging. Respondents emphasized the need for clearer, centralized communication to support consistent practice across studies and teams [4].

#### Receptivity and readiness

Despite workplace challenges, CRPs showed strong interest in continued professional learning and peer-based support (*n* = 44; 94%), including local peer groups (*n* = 38; 81%), national training networks (*n* = 30; 64%), and mentorship or community-building activities (*n* = 26; 55%). Overall, respondents demonstrated readiness to engage in enhanced training when supported by leadership and peers, provided clear expectations, resources, and organizational structures that integrate learning into daily workflows [4].

### Component 4: KT intervention design & exchange [2,3]

#### Workforce development programs for clinical research professionals

Survey responses offered clear direction for strengthening TWD programs through structured practice-based approaches that align training with competency-based approaches (what a learner must be able to demonstrate as skills or abilities). Respondents identified opportunities to reinforce foundational skills and support continued growth through accessible, well-organized learning pathways (across fundamental, skilled, and advanced experience levels). Collectively, survey results informed role-specific messaging, strategies for engaging stakeholders in shaping training progression, and the integration of CBE into organizational systems [9–11].

#### Knowledge broker

Findings reflected a need for roles that help translate guidance into daily practice across the complex and evolving research systems. CRPs expressed interest in timely clarification, consistent communication, and support in navigating evolving procedures, which are core functions of the KB within the RKTU framework [2–4]. KBs serve as translators and connectors between institutional expectations and frontline practice, adapting guidance to local contexts and supporting real-time problem-solving [2–4]. They can curate resources, coordinate updates, facilitate learning, and ensure training remains aligned with current expectations [2,3,5,16]. Because CRPs are strongly motivated by advancement, certification, and recognition, KB-guided programs can integrate clear pathways for growth [9,16,17]. Formalizing KB-type roles, such as Assistant/Associate Directors for TWD, within CTSA hubs or research practice partners strengthens KT/KTE capacity and supports TWD initiative sustainability [18–20].

#### Reception and champions

CRPs expressed openness to engaging in training when it was accessible and aligned with their professional goals [2,3]. Champions, who are experienced colleagues, supervisors, or role-specific leaders, play a meaningful part in reinforcing training uptake [2,3,5]. As influential insiders, champions help translate training into practice by modeling desired behaviors, encouraging peer participation, and reinforcing innovative approaches within daily workflows. Respondents noted that peer encouragement and visible local leadership increase confidence, teamwork, engagement, and adoption of new practices [2,3].

#### Types and timing of activities

CRPs indicated that training is most effective when delivered through layered and recurring activities offered at multiple time points. Respondents highlighted the value of combined formats, including workshops, mentoring opportunities, simulation, shadowing, and structured discussions, to reinforce concepts and responsibilities, as they evolve across experience levels and study phases [1,4,14,16]. In addition, respondents expressed interest in ongoing, easily accessible guidance and regular touchpoints that support continuous learning and practice application.

### Phased approach and outcome measures

Findings support a phased approach to TWD aligned with how KT unfolds over time within the RKTU framework [2–4]. This approach reflects CRP-identified needs for structured onboarding, accessible resources, and sustained professional support, while informing outcome measures across phases.

**Phase One (short-term)** focuses on addressing gaps and establishing infrastructure for consistent training and communication (*n* = 47; 100%) [4,5]. Strategies include communicating AHC expectations, assessing onboarding practices, and centralizing resources through platforms such as a TWD website and ListServ announcements [4,9,13,14]. Short-term outcomes include a structured TWD framework and pilot trainings that support clear role expectations, JIT resources, and workflow-relevant microlearning [2–4,6,9].

**Phase Two (mid-term)** focuses on expanding access through digital infrastructure and peer-supported learning (*n* = 42; 89%), including a CRP community platform (e.g., Microsoft Teams) (*n* = 33; 70%), discussion boards (*n* = 28; 60%), and structured mentorship programs *(n* = 26; 55%) [4,9,14,15]. Strategies include establishing local and national training networks, expanding applied, practice-based learning (e.g., onboarding, preceptorship, simulation), and offering expertise-level PD curricula in multiple formats, supported by regular feedback loops for refinement [16,21–23]. Coordination with human resources to align career pathways and roles, along with integration of engagement drivers into TWD programs, further supports implementation. Mid-term outcomes include increased training satisfaction, adoption, competency-based evaluation, and demonstrated application in practice, along with sustained mentorship structures and strengthened cross-site CRP networks.

**Phase Three (long-term)** focuses on scaling and embedding TWD within institutional systems [4]. Strategies include expanding access across roles and sites, formalizing career pathways, and strengthening shared governance and feedback structures to support sustainability [2–4,23,24]. Long-term outcomes include institutionalized processes, embedded career pathways, and recognition mechanisms, reflected in improved practice quality, workforce stability, retention, and sustained KTE across systems [2–4,9,10,17,23]. Ongoing KTE processes support continuous adaptation over time [2–4].

#### Formats and materials

Across roles, CRPs preferred blended or multimodal learning approaches that combine on-demand courses (*n* = 51; 93%) with opportunities for live interaction, including (1) live virtual trainings (*n* = 35; 64%); (2) hands-on practice including role-play and simulation (*n* = 33; 60%); and (3) in-person workshops and courses (*n* = 32; 58%). Respondents valued centralized materials (*n* = 34; 62%), available through easy-to-navigate online portals and learning platforms (*n* = 34; 62%), and directly applicable to their work, including concise guides, step-by-step instructions, and curated resource hubs. These preferences underscore the importance of training that is practical, streamlined, and immediately usable [5–7].

#### Motivators and engagement drivers

CRPs identified the number one motivator that encourages training engagement, includes having flexible program design formats, which are modular or JIT learning (*n* = 57; 98%). Other engagement drivers include: (1) learning with trusted colleagues (social motivation and peer-driven engagement) (*n* = 50; 86%); (2) administrative and organizational support (*n* = 50; 86%); (3) career advancement and skill transferability (*n* = 49; 84%); (4) financial incentives, visibility, recognition by leadership, and ability to influence or motivate others (*n* = 41; 71%). Other motivators include credentialing and formal qualifications (*n* = 40; 69%) and funding for specialty certifications and dissemination efforts (*n* = 39; 67%) (see Table 2) [4]. Respondents also pointed to the importance of manageable workloads, protected time, and increased salary. These factors help ensure that training engagement depends not only on perceived value but also on whether participation is feasible and supported within daily responsibilities [3,4].

**Table 2.** Preferred formats, community support, & engagement drivers (by role) Table 2 long description.

	Coordinator (*n* = 38)	Administrator (*n* = 16)	Investigator (*n* = 6)	Other (*n* = 5)
**Preferred training formats, materials, & resources**				
Online portals/learning platforms	●●	●●●	●	●●●
On-demand courses	●●●	●●●	●●●	●●
Live in-person workshops/courses	●●	●●	●	●●
Live virtual workshops/courses	●●●	●	●●	●●●
Webinars/other virtual events	●●	●●	●	●
Role-play simulation	●●●	●●	●	●●●
Shadowing	●●	●	●●	●●●
Conferences/symposiums	●●	●●	●	●
Printed manuals/reference sheets	●●	●●	●	●●
Centralized training resources/guides	●●●	●●	●	●●●
**Clinical research professionals community group activities & support**				
Digital infrastructure & tools	●●●	●●●	●	●●●
Centralized resource sharing	●●●	●●●	●	●●●
Discussion boards (peer Q & A/sharing best practices)	●●●	●●	●	●●
People-based groups & networks	●●●	●●●	●●●	●●●
PD groups	●●●	●●●	●	●●●
RPN/National training networks	●●●	●●●	●	●
Mentorship & peer support programs	●●	●●●	●	●●●
Communications, events, & PD messaging	●●●	●●●	●●●	●●●
ListServ, newsletters, event announcements	●●●	●●●	●	●●●
CEU/CME program communication	●●	●●	●	●●●
**Motivators & engagement drivers**				
Career advancement & skill transferability	●●●	●●●	●●	●●●
Funded credentialing & formal qualifications	●●●	●●●	●	●●●
Incentives, visibility, & influence	●●●	●●	●●	●●●
Structural & Organizational support	●●●	●●	●●●	●●●
Program design, flexibility, & accessibility	●●●	●●●	●●●	●●●
Social motivation & peer-driven engagement	●●●	●●●	●●●	●●●

Top Need = ●●● (67% – 100%); Moderate Need = ●● (34% – 66%); Lowest Need = ●7 (0% – 33%)

Sp. = Specialist; CE = Continuing Education; PD = Professional Development; RPN = Research Professionals Network; Q & A = Questions & Answers

*Note:* Other includes reflections from Data Specialist, Regulatory Analyst, and Research Assistants. Dot priorities represent aggregate onboarding and professional development needs, with role-level adjustments where coded responses show stronger emphasis. Because roles have small Ns, differences should be interpreted cautiously-as guidance for program design rather than statistical inferences.

#### Intensity of interaction

Respondents indicated that sustained engagement, across short-, mid-, and long-term intervals supports deeper KU [2,8]. Findings highlight the importance of recurring group-based activities, such as mentorship programs, interdepartmental collaborations, certification study groups, publishing groups, and shared governance opportunities, which build connection and support ongoing development [24] (see Table 3). Together, these layered touchpoints reflect the RKTU expectation that ongoing interaction strengthens conceptual understanding, promotes procedural integration, and supports structural use [4,8].

**Table 3.** Outcomes and interaction intensity (by role) Table 3 long description.

Role	Short-term outcomes	Mid-term outcomes	Long-term outcomes	Interaction intensity
**Coord (*n* = 38)**	Structured onboarding pathway with centralized, role-specific guidance and checklists	Applied learning through simulation, shadowing, peer mentoring, and workflow-focused cohorts	Competency-based advancement pathways with recognition and role progression (career ladder)	Integrated repeated touchpoints (e.g., regular Q&A sessions, periodic workshops, recurring simulation refreshers)
**Admin (*n* = 16)**	Centralized digital resource hub and SOP library to support consistency across departments	Cross-department workflow alignment, leadership development, mentoring, and MS Teams communities	Institutionalized recognition, leadership pathways, and refined career ladders	Recurring communication loops (e.g., regular updates, periodic alignment and leadership meetings)
**Assist (*n* = 3)**	Foundational SOP training and task- level walkthroughs (from identified priority areas)	Guided simulation of visit flow and scenario-based operational learning	Microcredentials or milestones linked to advancement and role transition	Frequent, structured touchpoints (e.g., regular check-ins and refreshers)
**Reg** **(*n* = 1)**	Targeted efforts toward transparent and consistent regulatory workflows	Advanced simulation focused on deviations, safety reporting, and CAPAs	Formalized certification and specialization pathways	Recurring advanced regulatory touchpoints (e.g., focused CAPA reviews and regulatory deep dives)
**Data Sp.** **(*n* = 1)**	Data quality, governance, and informatics practices integrated into onboarding	Advanced monitoring, analytics, and data-communication workshops	Data stewardship certifications and recognition	Periodic calibration and analytics sessions (e.g., data quality clinics and monitoring reviews)
**Invest (*n* = 6)**	Investigator-focused SOPs, IRB navigation, and expectation-setting resources	Team science onboarding, investigator responsibility, and communication workshops	Integration of research and training expectations into performance review structures	Recurring investigator touchpoints (e.g., workshops, regulatory updates, and team science forums)

Coord = Coordinator; Admin = Administrator; Assist = Assistant; Reg = Regulatory Analyst; Data Sp. = Data Specialist; Invest = Investigator; Q&A = Question and Answer; SOP = Standard Operating Procedure; MS = Microsoft; CAPA = Corrective and Preventive Action; IRB = Institutional Review Board.

### Component 5: Knowledge utilization evaluation [2–4]

#### Conceptual use

CRPs demonstrated a clear understanding of key concepts, expectations, and organizational structures shaping clinical research practice [2,3]. Their responses suggest a shared baseline understanding that aligns with nationally recognized competency frameworks, including the Joint Task Force (JTF) for Clinical Trial Competency [15] and International Association of Clinical Research Nurses (IACRN) standards [25].

#### Effort to use

CRPs described personal initiatives looking for ways to strengthen skills or better align learning with career advancement and certification goals, often through self-initiated and personal financial investment. These behaviors demonstrated the effort CRPs invest to use knowledge in practice, even in the absence of consistent systems, guidance, or formal training infrastructure [2,3].

#### Procedural use

CRPs described applying what they learned from peers to their everyday work procedures [2–4]. Respondents referenced creating checklists, standardizing task sequences, monitoring documentation quality, and using established tools to maintain accuracy and compliance in research activities. These practices reflect localized procedural use that has developed through experiences and necessity, rather than through formalized training structures [2–4].

#### Structural use

Structural use refers to organization-level supports that embed knowledge into routine practice [2–4]. Qualitative findings described limited but existing structures, including shared systems and department-specific training resources. Respondents indicated readiness for stronger coordination, clearer structures, and better alignment of policies to support long-term KU [2–4]. Addressing these structural conditions is critical for TWD acceptance and sustainability [8] and provides a foundation for structured onboarding and progression across experience levels [2,3].

## Discussion

This Special Communication applies an RKTU-informed approach to understand how CRPs experience training and use it in practice, extending D&I science by showing how knowledge, context, and systems influence uptake and KU. Findings suggest the challenge is not motivation, but applying training in complex work environments, demonstrating the need for more relevant, accessible, and integrated TWD.

Several CTSA initiatives illustrate this alignment. Multimodal models, such as Duke University’s integrated onboarding, mentoring, and CBE, support progressive skill development [14]. Simulation-based learning further enables CRPs to practice complex tasks and build confidence before applying skills in real-world settings [16]. These approaches reflect a clear preference for APBL that supports immediate use.

Findings demonstrate the importance of centralized training infrastructure. Shared platforms (e.g., DIAMOND portal and Georgia CTSA catalogs) and cross-institutional initiatives (e.g., Research Professionals Network) reduce duplication, improve access, and support continuous learning [21,22,26]. These models provide practical examples for scaling TWD while maintaining local relevance and advancing translational science through collaborative, cross-institutional approaches that support the development of generalizable solutions [27].

Shared governance is another critical component. This can be implemented through CRP advisory councils, peer-led groups, and co-designing training initiatives that allow frontline staff to inform program development [24]. These approaches support workforce ownership and ensure training remains responsive to evolving needs [24].

Professionalization of the CRP workforce requires support for both certification and academic advancement. Recognizing and incentivizing both may strengthen career progression, retention, and engagement.

The role of KBs emerged as central to supporting KT/KTE and workforce engagement. In this study, KB functions were reflected in individuals who bridge training programs, workflows, and institutional priorities, often TWD leads, program managers, or clinical and educational leaders with system-level insight [18–20]. KB roles can improve coordination, alignment, and sustained KU [2–4].

Building on this, the findings also support hybrid leadership models that integrate administrative and practice-based expertise. KBs (or “TWD administrators”) oversee training design, collaboration, and alignment with AHC and CTSA missions. Expanding joint administrator-faculty appointments, particularly for doctoral-prepared leaders, may strengthen program leadership, improve access to funding, and better integrate practice-based expertise into Academic Practice Partnership TWD initiatives [18–20].

More broadly, this study demonstrates how D&I frameworks can be used not only to evaluate training systems but to design them. Aligning TWD with RKTU principles offers an evidence-informed approach to strengthening the clinical research workforce and advancing translational science priorities [8].

### Lessons learned

Training should focus on real-world tasks, using APBL approaches such as case-based learning, simulation, and repeated skill-building. TWD must fit within daily workflows, with JIT access and dedicated time for learning. Sustained impact relies on mentorship, communities of practice, and workforce leaders who reinforce learning over time. Scaling TWD requires coordination across programs and institutions, using shared resources to reduce duplication while allowing local adaptation. Overall, effectiveness depends not just on content but on how well training fits within the systems where CRPs work.

### Limitations

This study reflects CRP experiences within a single AHC, which may limit generalizability. Some roles were represented by few respondents, limiting role-specific insights. Findings are based on self-reported perceptions rather than direct observation, though these perspectives are central to understanding KU. Finally, dissemination-informed approaches may not fully address broader structural barriers to adoption [8].

### Future directions

Future work will implement and evaluate the role-specific pilot initiatives identified to assess feasibility and workforce outcomes. Additional research is needed to examine how TWD strategies influence broader translational outcomes, including study quality, efficiency, and workforce sustainability. Frameworks (e.g., Translational Benefits Model) may support evaluation of these impacts [28].

## Conclusion

Integrating D&I science into TWD provides a practical pathway to improving training relevance, engagement, and use. This study shows that training effectiveness depends on how well it aligns with real-world practice and organizational systems. Embedding TWD within workflows can support sustained KU and strengthen the clinical research workforce across CTSA hubs and AHCs.

## Supporting information

10.1017/cts.2026.10789.sm001 Carey et al. supplementary material 1 Carey et al. supplementary material

10.1017/cts.2026.10789.sm002 Carey et al. supplementary material 2 Carey et al. supplementary material

## Supplementary material

The supplementary material for this article can be found at https://doi.org/10.1017/cts.2026.10789.

## Figure 1. Long description

This framework describes factors that influence knowledge utilization (KU) in developing the translational workforce development (TWD) of clinical research professionals (CRPs). The five components include: (1) Knowledge Source (e.g., credible source, rigorous evidence, audience-specific information, and authentic voice; (2) Knowledge Transfer (KT) (e.g., compelling idea, tailoring, and relevance); (3) Workplace Context (e.g., organizational structures, workflows, ongoing change, receptivity, and readiness); (4) Intervention and Knowledge Transfer and Exchange (KTE) (e.g., phased approaches, knowledge brokers (KB), champions, activities, formats, and materials, motivators, engagement drivers, and interaction intensity); and (5) KU Evaluation (e.g., conceptual, effort, procedural, and structural use). One-way KT and bidirectional exchange between learners and program administrators lead to phased outcomes that strengthen infrastructure, career pathways, and continuous improvement.

Navigate back to Figure 1..

## Table 1. Long description

Table 1 presents priority training needs for clinical research professionals’ workforce roles: coordinators (n=38), administrators (n=16), investigators (n=6), and other personnel (n=5). Priority training needs include: (1) onboarding and (2) ongoing professional development (PD). Onboarding topics include institution-specific requirements and standard operating procedures, regional resources, mentoring, precepting, simulation, and standardized competency-based education. Ongoing PD includes leveled priority training needs based on the Joint Task Force for Clinical Trial Competency Framework domains. Dot ratings indicate the priority level of training based on role responses. The highest priorities across roles included onboarding, simulation-based learning, and ongoing PD, including prioritized training in study and site management, data management and informatics, leadership, and communication skills.

Navigate back to Table 1..

## Table 2. Long description

Table 2 summarizes preferred training formats, community support needs, and engagement drivers by role. Preferred training formats include on-demand courses, live learning, role-play simulations, centralized training resources, and online learning platforms. Clinical Research Professionals (CRPs) community group activities and support include digital infrastructure and tools, people-based groups, and networks. Motivators and engagement drivers include career advancement and skill transferability, funded opportunities, organizational support, and peer-driven engagement.

Navigate back to Table 2..

## Table 3. Long description

Table 3 presents recommended outcomes and engagement interaction intensity by Clinical Research Professionals (CRPs) role. For Coordinators: Progress from developing and evaluating structured onboarding with centralized role-specific guidance and checklists to applied learning through simulation, mentoring, and competency-based career advancement. Administrators: centralized digital resource hubs, standard operating procedure (SOP) libraries, cross-departmental training and systems, and career ladders. Assistants: SOP training, simulations, and microcredentials. Regulatory Analysts: regulatory workflows, simulation, and certification pathways. Data Specialists: Data quality onboarding, analytics training, and data stewardship certification. Investigators: SOPs, IRB navigation, team science, and research expectations in performance reviews. All roles recommend recurring communication, workshops, mentoring, forums, and structured touchpoints designed to reinforce professional growth.

Navigate back to Table 3..
