# Supplementary material for: Designing translational workforce development programs using the research knowledge and transfer utilization framework
Source: J Clin Transl Sci. 2026 Jul 13;10(1):e136. doi: 10.1017/cts.2026.10789 (PMC13458298; doi:10.1017/cts.2026.10789)
Supplement: Supplementary file 1 [file S2059866126107894sup002.pdf]

Intro, Demographic, Training and Education

We want to learn how we can better support your work as a research professional (e.g., scientists, analysts, coordinators, nurses).

Please take this survey to share your training needs, challenges, and support preferences. Your feedback helps us tailor programs to suit your needs and enhance your confidence at work.

Thank you for your time and dedication. We look forward to your insights!

If you have any questions or concerns with this survey, please reach out to Alexandria Carey alexandria.carey@ufl.edu

1 of 6 Demographic Information:

What is your job title?

____

How many years of experience do you have in the research field?

- ○ Less than 1 year
- ○ 1-2 years
- ○ 3-5 years
- ○ 6-10 years
- ○ More than 10 years

How long have you been in your current role?

- ○ Less than 1 year

- 1-2 years
- 3-5 years
- 6-10 years
- More than 10 years

Which location & department do you work in?

____

2 of 6 Training and Education:

a. Onboarding training (during your first year on the job)

Did you receive onboarding for your current role?

- Yes
- No
- Not sure
- Other (please specify): __________

Was your onboarding enough?

- Yes
- No
- Not sure
- Other (please specify): __________

What training would have improved your onboarding?

____

Did your onboarding include: (Select all that apply)

- ☐ One-on-one shadowing
- ☐ Self-paced learning including online training
- ☐ Instructor-led education including lectures / ppt presentations
- ☐ Online or verbal (tabletop) scenario-based education
- ☐ Simulation-based / active role-play training
- ☐ Other (please specify): ____

b. Continued professional development (training and education after your first year on the job)

Since onboarding, have you received additional training specific to your current role?

- ○ Yes
- ○ No
- ○ Not sure
- ○ Other (please specify): ____

If yes, what type of training?

- ○ Online, internal to your current position
- ○ Online, external to your current position
- ○ Professional conferences
- ○ Research conferences
- ○ Other (please specify): ____

Was the training paid for by you or your department?

- ○ Department paid
- ○ Self-pay
- ○ None, all training has been free
- ○ Both my department and I paid
- ○ Other (please specify): ____

How confident are you in performing essential tasks?

- ○ 5 Extremely Confident
- ○ 4 Moderately Confident
- ○ 3 Neutral
- ○ 2 Not Very Confident
- ○ 1 Extremely Not Confident

How satisfied are you with the quality of your training for your current role?

- ○ 5 Extremely Satisfied
- ○ 4 Satisfied
- ○ 3 Neutral
- ○ 2 Dissatisfied
- ○ 1 Extremely Dissatisfied

Which areas of research do you feel you need more training in? (Select all that apply)

- ☐ Protocol Adherence
- ☐ Research Administration / Subject Management
- ☐ Documentation / Documents
- ☐ Good Clinical Practice
- ☐ Human Subject Protection
- ☐ Institutional Requirements
- ☐ Job Specific & Department Requirements
- ☐ Pre-Study Start-Up
- ☐ Recruitment & Retention
- ☐ Regulatory Environment
- ☐ Data Monitoring
- ☐ Safety Monitoring
- ☐ Study Closure
- ☐ Research Innovation
- ☐ Other (please specify): ______

Protocol Adherence:
Please select from the following: (Select all that apply)

- ☐ Coordination of clinical treatment
- ☐ Coordination of study visits
- ☐ Coordination of follow-up care

- ☐ Collection, processing, and shipping of laboratory specimens
- ☐ Standard Operating Procedures
- ☐ Other (please specify): ______

Research Administration / Subject Management:
Please select from the following: (Select all that apply)

- ☐ Billing Resolution
- ☐ Managing study finances, including resolving billing issues for study participants
- ☐ Other (please specify): ______

Documentation / Documents:
Please select from the following: (Select all that apply)

- ☐ EPIC documentation
- ☐ OnCore
- ☐ Paper / Study Binders
- ☐ Regulatory – Accrual, Adverse Events (AEs), Deviations, etc.
- ☐ Maintenance of study source documents
- ☐ Deviation Tracking Table
- ☐ Adverse Events Table
- ☐ Trial Master File
- ☐ Other (please specify): ______

Good Clinical Practice:
Please select from the following: (Select all that apply)

- ☐ Ethical Conduct of Research
- ☐ Informed Consent
- ☐ Protocol Adherence
- ☐ Institutional Review Board (IRB)/Ethics Committee Oversight
- ☐ Investigator Qualifications and Responsibilities
- ☐ Data Integrity and Confidentiality
- ☐ Quality Assurance and Quality Control
- ☐ Safety Reporting
- ☐ Compliance with Regulations
- ☐ Record Keeping and Reporting
- ☐ Other (please specify): ______

Human Subject Protection:
Please select from the following: (Select all that apply)

- ☐ Safety of clinical research subjects
- ☐ Proper reporting of adverse events
- ☐ Ethics and Requirements
- ☐ Other (please specify): ______

Institutional Requirements:
Please select from the following: (Select all that apply)

- ☐ OnCore
- ☐ MyTraining
- ☐ EPIC
- ☐ Human Subject Participant Payment System
- ☐ UF Health Intervention Review Board (IRB)
- ☐ Clinical and Translational Science Institute (CTSI) resources
- ☐ Other Technology (please specify): ______
- ☐ Other (please specify): ______

Job Specific & Department Requirements:
Please select from the following: (Select all that apply)

- ☐ On-boarding new team members
- ☐ Coordinating communication between subjects, researchers, IRB, sponsors, and healthcare professionals
- ☐ Role-Playing
- ☐ Workflow
- ☐ Communication
- ☐ Teamwork/Collaboration
- ☐ Team Science
- ☐ Conflict Resolution
- ☐ Managing Up
- ☐ Networking
- ☐ Mentoring
- ☐ Publishing
- ☐ Training Opportunities
- ☐ Where to find resources
- ☐ Resources
- ☐ Streamlining
- ☐ Certifications
- ☐ Other (please specify): ______

Pre-Study Start-Up:

Please select from the following: (Select all that apply)

- ☐ Facilitate Contract
- ☐ Budget Preparation / Negotiations
- ☐ Cost of Service Requests
- ☐ REDCap study set up
- ☐ Cohort Discovery (i2B2)
- ☐ Grants
- ☐ Coordination of pre-study, initiation, and monitoring visits
- ☐ Other (please specify): ____________

Recruitment & Retention:

Please select from the following: (Select all that apply)

- ☐ Subject screening
- ☐ Subject recruitment
- ☐ Subject enrollment
- ☐ Other (please specify): ____________

Subject recruitment:

Please select from the following: (Select all that apply)

- ☐ Fliers
- ☐ Social Media
- ☐ HealthStreet
- ☐ Recruitment Center
- ☐ UF Studies website
- ☐ Recruitment using clinical staff
- ☐ Other (please specify): ____________

Regulatory Environment:

Please select from the following: (Select all that apply)

- ☐ Submissions to regulatory authorities [e.g., IRB, Food and Drug Administration (FDA), etc.]
- ☐ Creating Corrective and Preventive Actions (CAPAs)

- [ ] ClinicalTrials.gov compliance
- [ ] Regulatory documentation development and management
- [ ] Completion of case report forms (paper & electronic data capture)
- [ ] Coordination of pre study, initiation, and monitoring visits
- [ ] Risk-based monitoring
- [ ] Maintenance of drug accountability documentation
- [ ] Managing data queries
- [ ] Other (please specify): ______

Submissions to regulatory authorities:

Please select from the following: (Select all that apply)

- [ ] Single IRB (SIRB)
- [ ] Western IRB (WIRB)
- [ ] Local IRB
- [ ] Investigational Drugs and Devices (IND / IDE)
- [ ] Other (please specify): ______

Data Monitoring:

Please select from the following: (Select all that apply)

- [ ] REDCap study monitoring
- [ ] HiPerGator
- [ ] Other (please specify): ______

Safety Monitoring:

Please select from the following: (Select all that apply)

- [ ] Serious Adverse Event and Adverse Event (SAE/AE) reporting
- [ ] SAE/AE adjudication
- [ ] Other (please specify):

Study Closure:

Please select from the following: (Select all that apply)

- ○ REDCap study closure
- ○ Other (please specify): ________

Research Innovation:

Please select from the following: (Select all that apply)

- ☐ Artificial Intelligence (AI) in research
- ☐ Other (please specify): ________

Challenges and Barriers

3 of 6 Challenges and Barriers:

What are the biggest challenges you encounter in your current position as a research professional?

____

Are there any institutional or organizational barriers that hinder your effectiveness as a research professional?

____

What resources or support do you feel are lacking in your current position?

____

Preferences for Training Delivery

4 of 6 Preferences for Training Delivery:

What types of training methods or formats do you prefer (e.g., workshops, online courses, on-the-job training)? (Select all that apply)

- ☐ Virtual “live” workshops
- ☐ In-person “live” workshops / courses
- ☐ Online “on-demand” courses
- ☐ Role-play / Simulation opportunities
- ☐ Other (please specify): ____

How do you prefer to receive training materials and resources (e.g., printed manuals, online portals)? (Select all that apply)

- ☐ Printed manuals / printed reference sheets
- ☐ Online portals
- ☐ Other (please specify): ____

What factors would motivate you to participate in training opportunities (e.g., incentives, recognition, career advancement)? (Select all that apply)

- ☐ Incentives
- ☐ Recognition
- ☐ Career Advancement
- ☐ Continuing Education towards Certification
- ☐ Other (please specify): ____

Research Professional Development Community

A research professional development community is a network or group of individuals and organizations dedicated to supporting the growth and advancement of research professionals. Such communities provide resources, opportunities, and forums for research professionals to enhance their skills, knowledge, and careers.

5 of 6 Research Professional Development Community:

a. Are you currently a part of a research professional development community?

- ◯ Yes
- ◯ No, but I want to be
- ◯ No, I am not interested in being a part of a research professional development community

How well do each of the following statements represent how you feel about this community?

	Completely	Mostly	Somewhat	Not at all
I put a lot of time and effort into being part of this community.	◯	◯	◯	◯
If there is a problem in this community, members can get it solved.	◯	◯	◯	◯
Members of this community care about each other.	◯	◯	◯	◯

If you are not interested in being a part of a research professional development community, please contact Alexandria Carey alexandria.carey@ufl.edu to be removed from the ListServ.

b. Fostering professional growth created by community opportunities for networking, collaboration, and ongoing learning is one of our top priorities.

Which current position support would you use? (Select all that apply)

- ☐ Community Interaction
- ☐ Professional Development
- ☐ Recognition and Awards
- ☐ Resources and Documentation
- ☐ Publications and Media

- [ ] Associations and Networks

Community Interaction:
Please select from the following: (Select all that apply)

- [ ] Discussion Boards (Q&A, sharing best practices)
- [ ] Role-specific channels
- [ ] Town Halls
- [ ] Journal Clubs
- [ ] Other (please specify): ______

Professional Development:
Please select from the following: (Select all that apply)

- [ ] Training Announcements
- [ ] Certification Study Groups
- [ ] Mentorship Programs
- [ ] Continuing Education Programs
- [ ] Webinars and Virtual Events
- [ ] Collaborative Research Initiatives
- [ ] Research Professionals Network (national training)
- [ ] Other (please specify): ______

Recognition and Awards:
Please select from the following: (Select all that apply)

- [ ] Digital Badges
- [ ] Awards and Recognition Board
- [ ] Microcredentialing
- [ ] University of Florida (UF)-related Certifications
- [ ] Other (please specify): ______

Resources and Documentation:
Please select from the following: (Select all that apply)

- [ ] Centralized Resources (including QuickLink documentation & Toolkits)

- [ ] Research Professionals Contact List (with photos)
- [ ] Other (please specify): ____________

Publications and Media:
Please select from the following: (Select all that apply)

- [ ] Publications
- [ ] Newsletters
- [ ] Social Media
- [ ] Other (please specify): ____________

Associations and Networks:
Please select from the following: (Select all that apply)

- [ ] Professional Associations
- [ ] Online Forums and Networks
- [ ] Other (please specify): ____________

Suggestions for Improvement

6 of 6 Suggestions for Improvement:

Do you have any suggestions for improving the training and professional development opportunities for research professionals on campus?

____

Is there anything else you would like to add regarding your training needs or experiences as a research professional?

____

©University of Florida
Gainesville, FL 32611
Terms of Use

Powered by Qualtrics
